# Analysis of Early Postoperative Pain in the First and Second Knee in Staged Bilateral Total Knee Arthroplasty: A Retrospective Controlled Study

**DOI:** 10.1371/journal.pone.0129973

**Published:** 2015-06-11

**Authors:** Jiuyi Sun, Lintao Li, Shuai Yuan, Yiqin Zhou

**Affiliations:** 1 Department of orthopedics, PLA 455 Hospital, Shanghai, China; 2 Department of orthopedics, The Second Affiliated Hospital of Second Military Medical University, Shanghai, China; College of Medicine, TAIWAN

## Abstract

**Objective:**

A retrospective analysis of early postoperative pain in the first and second knee in staged bilateral total knee arthroplasty (TKA) to provide a clinical evidence for the change of analgesic strategy.

**Methods:**

From January 2009 to January 2013, 87 cases which meet the inclusion criterion were retrospectively reviewed. In stage TKA, the postoperative pain in the first and second knee at 24h, 48h, 72h after operation were compared using the visual analogue scale (VAS) score in the rest and maximum knee flexion position. The difference in pain scores (ΔVAS) was also compared between the second and first knee at different time intervals (less than 6 months, 6-12 months, more than 12 months).

**Results:**

The VAS scores in the second knee were significantly higher than those in the first knee at 24h, 48h after surgery, but with no difference at 72h. The ΔVAS in the group of less than 6 months was significantly higher than of those more than 6 months, and there was no difference in ΔVAS between group of 6-12 months and group of more than 12 months.

**Conclusions:**

Patient receiving staged bilateral TKA experiences greater postoperative pain within 48h after operation in the second knee than in the first knee, which can provide a clinical evidence to enhance the analgesic strategy in the second operation of the staged bilateral TKA. And for the management of postoperative pain in staged bilateral TKA, it’s better to recommend that the interval between two operations should be more than 6 months, which may reduce the postoperative pain in the second knee, improve patient satisfaction, and speed up patient‘s rehabilitation process.

## Introduction

As a conventional surgery for the treatment of end-stage knee osteoarthritis, total knee arthroplasty (TKA) is used for relieving pain in patients and improving their knee functions. The surgery has been widely used around the world, and the annual cases of global TKA also increased sharply [1, 2]. Osteoarthritis of the knee is often associated with bilateral involvement; some investigators have reported that approximately 18.6% of the patients treated with TKA need a bilateral knee replacement [3]. Choosing simultaneous or staged bilateral TKA is still a controversial issue. Some studies showed that simultaneous bilateral TKA can reduce the in-hospital expense and recovery time, etc.[4–11] However, simultaneous bilateral TKA may have increased the incidence of gastrointestinal discomfort, deep vein thrombosis, pulmonary embolism, fat embolism, cardiovascular disease and mortality. Therefore, more investigators believed that a staged bilateral TKA is a safer approach than the bilateral TKA [5, 12–17].

Postoperative pain of TKA is the most commonly reported complaint that surgeons face [18]. Postoperative pain is also one of the important factors affecting patients’ postoperative knee function. It not only leads to patients’ dissatisfaction with the treatment results, but also hinders their postoperative knee exercise, ability to walk, and delays their hospital discharge and rehabilitation [19]. Although there have been extensive in-depth research on perioperative analgesia for joint replacement, including preemptive analgesia, intraoperative injection of analgesic cocktails, and a multi-modal analgesia [20–23], there are few studies focused on the postoperative pain in the first and second knee in staged bilateral TKA. Although the second knee in staged bilateral TKA also belongs to the category of initial knee replacement and we have generally managed the pain in accordance with conventional pain management protocol for initial knee replacement, it’s common that patients after the second operation need an intensive analgesic solution due to a greater pain than that after the first TKA. Therefore, we carried out this study to explore this phenomenon by analyzing the postoperative pain in staged bilateral TKA and found whether there is a difference in pain severity between the two operations and its pattern, thus providing evidence for choosing clinical analgesic strategies.

## Materials and Methods

### Ethics statement

The study was approved by the local ethics committee (Ethics committee of PLA 455 Hospital), and written informed consent was obtained.

### General Information

This retrospective controlled study included all eligible patients who underwent staged bilateral total knee arthroplasty in our hospital between January 2009 and January 2013. Inclusion criteria: patients who were preoperatively diagnosed as severe bilateral knee osteoarthritis and failed to respond to conservative treatments, with an American Society of Anesthesiologists (ASA) score of I or II, generally in good condition and suitable for staged bilateral total knee arthroplasty, with no mental and neurological disorders, and with no alcohol or drug addiction. Exclusion criteria: patient with a diagnosis other than osteoarthritis (including rheumatoid arthritis, pigmented villonodular synovitis, synovial chondromatosis, etc.), previous history of knee surgery or infection, underwent revision surgery during follow-up due to infection or other causes, with a history of pain syndrome, blood transfusion in perioperative period of either knee joint replacement surgery, difference in preoperative VAS score between bilateral knee≥20, and those who cannot understand and complete the VAS test. In this study, we used 0–100 VAS for measurement, and these VAS scores were collected in a face-to-face way. There were 87 patients who met the above criteria and had completed follow-up data. This retrospective analysis was conducted based basis on the clinical data of these 87 patients, of which 31 were males and 56 were females. The average age was 68.3 ± 8.2 years. And in 26 cases the two operations were performed within an interval of less than 6 months, 34 cases with an interval of 6–12 months and 27 cases with an interval of more than 12 months.

### Operation and Rehabilitation Method

All operations were performed under general anesthesia. Operations on both sides of each patient used the same surgical technique, the same implant, the same analgesic protocol, and the same rehabilitation strategy. All surgeries were performed by an experienced joint surgeon. The posterior cruciate ligament-retaining implants (Genesis II; Smith & Nephew, Memphis, TN, USA) were used in all cases. Osteophytes on the edge of the patella were routinely trimmed in all patients, but no patient underwent patellar arthroplasty. A mixture analgesic, which comprised of the combination of morphine, ropivacaine, compound betamethasone and epinephrine, was used during the operation [23]. The analgesic was injected at the medial, lateral and posterior knee joint capsule and the attachment of pes anserinus tendon, and ropivacaine was subcutaneously injected at the incisal edges during wound closure. Implants were fixed with bone cement and no drainage was placed after the operation. A combination of celecoxib and tramadol hydrochloride was used as postoperative analgesic (use other NSAIDs, if a patient was allergic to sulfonamide). All patients walked with a walking-aid 24 hours after the surgery and conducted muscle and range of motion exercises progressively.

### Outcome Measures

The VAS scores of two groups were compared after 24h, 48h and 72h (0 = no pain, 100 = worst pain). VAS scores were evaluated with the operated limb in rest position and in maximum flexion position respectively, while the contralateral limb was always in the rest position. The time intervals between both operations for all patients were greater than 3 months. And we observed the impact of different time intervals (less than 6 months, 6–12 months, more than 12 months) on the difference in pain severity between the second and first knee at 24h after operation (ΔVAS = VAS scores in the second knee—VAS scores in the first knee), including ΔVAS in rest position and ΔVAS in maximum flexion position.

### Statistical Analysis

Statistical analysis was performed using SPSS software, version 18.0. Early postoperative pain scores (VAS) between the both operations were compared using paired t-test, and difference in pain severity (ΔVAS) between the second and first knee in different time intervals was compared using one-way analysis of variance (ANOVA) and followed post hoc test (LSD-t test). P <0.05 was considered statistically significant.

## Results

This retrospective study enrolled a total of 87 eligible patients, 31 of which were male and 56 were female. The average age at study enrollment was 68.3±8.2 years and the average Body mass index (BMI) was 28.4±4.3. 23 of these patients were with an ASA score of I and the other 64 were with ASA score of II. There was no significant difference between the first and second knee in terms of patients’ VAS scores in rest position and in maximum flexion position and preoperative ROMs. And there was also no significant difference in the total operation time, tourniquet time and blood loss between the first and second surgery in staged TKA (Table 1).

**Table 1:** Comparison of the general information between the first and second surgery in staged TKA.

Item	First TKA (n = 87)	Second TKA (n = 87)	P Value
Preoperative VAS score in rest position	12.8±6.5	13.2±6.8	P = 0.460
Preoperative VAS score in maximum flexion position	76.9±6.5	76.8±8.7	P = 0.938
Preoperative ROM	101.6±15.2	102.3±13.8	P = 0.079
Operation time (min)	85.6±17.3	86.7±14.5	P = 0.119
Tourniquet time (min)	61.3±7.8	62.9±8.6	P = 0.053
Intraoperative blood loss (mL)	120.6±13.2	124.5±17.1	P = 0.081

**Fig 1:**
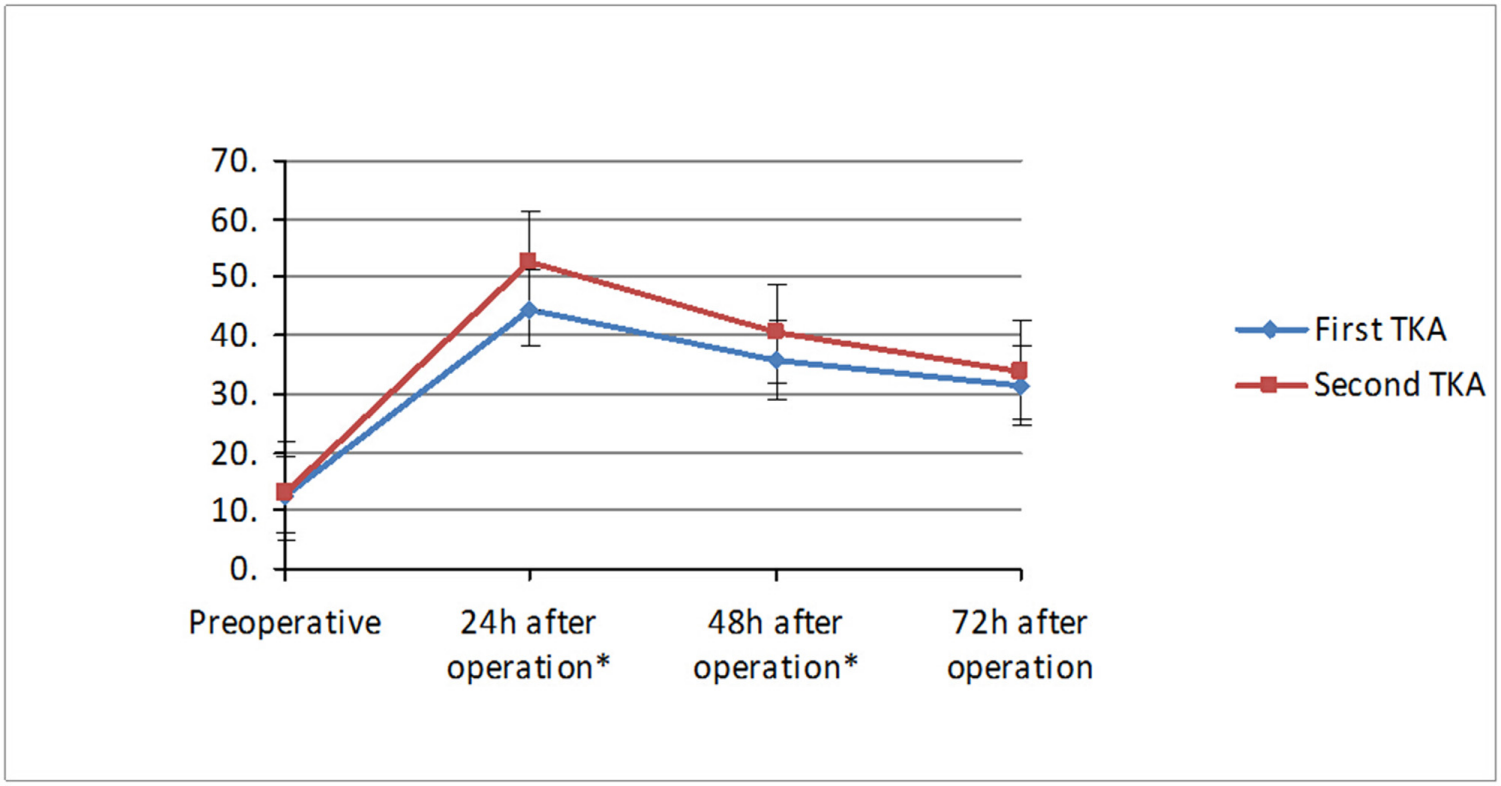
Comparison of VAS scores in rest position between two TKA surgeries. *P<0.05.

**Fig 2:**
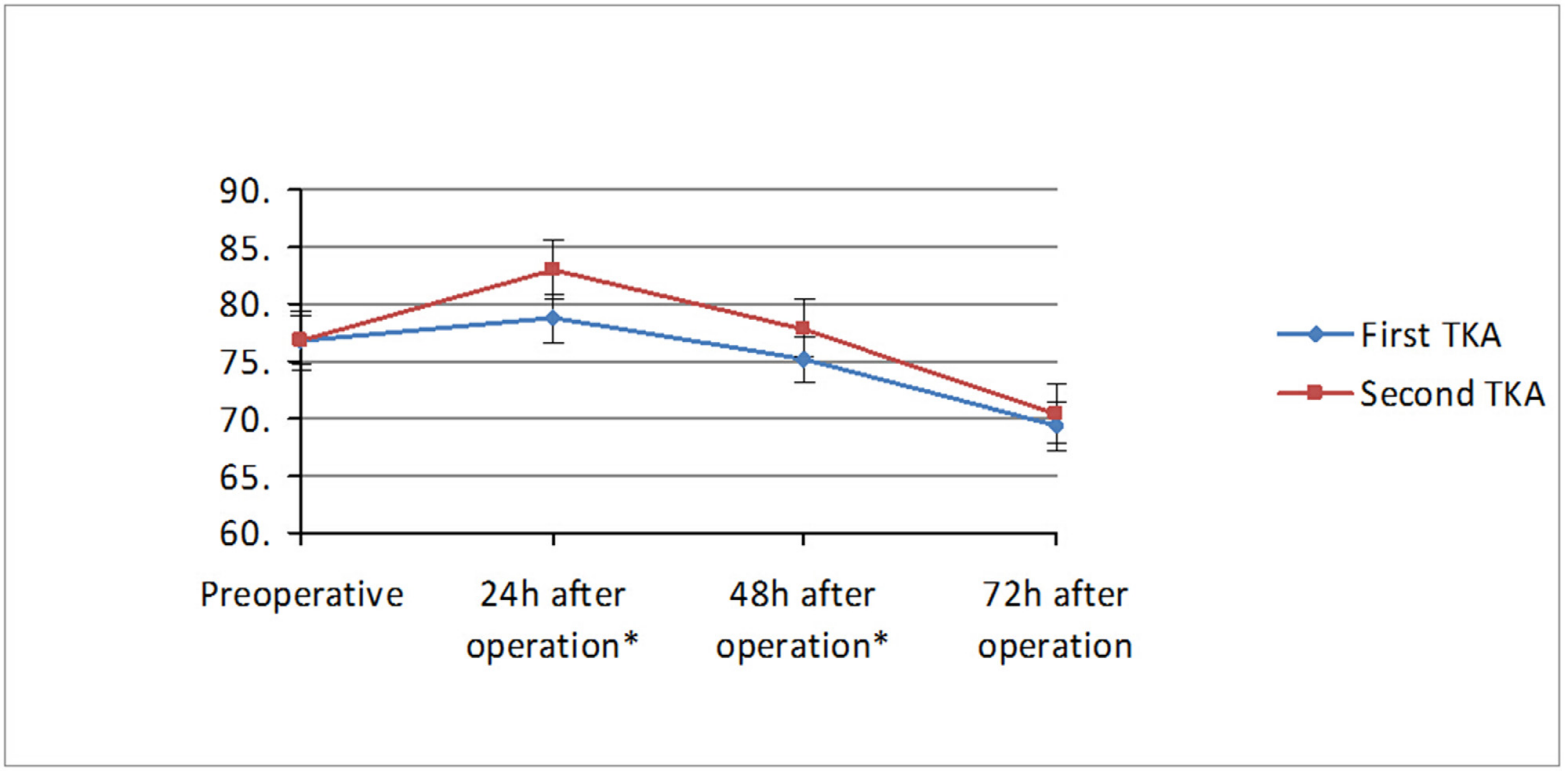
Comparison of VAS scores in maximum flexion position between two TKA surgeries. *P<0.05.

Comparison results of VAS scores in the first and second knee at 24h, 48h and 72h are shown in Table 2, Figs 1 and 2. The paired t-test results showed that the preoperative VAS scores in rest position and in maximum flexion position between the first and second knee had no significant difference, but the VAS scores in the second knee (both in rest position and in maximum flexion position) were significantly higher than in the first knee at 24h, 48h after surgery, but with no significant difference at 72h.

**Table 2:** Comparison of VAS scores between the first and second surgery in staged TKA.

VAS scores (in rest position)	preoperative	24h after operation*	48h after operation *	72h after operation
First TKA	12.8±6.5	44.6±10.0	35.7±9.6	31.3±11.2
Second TKA	13.2±6.8	52.8±8.1	40.3±8.9	34±10.0
P values	0.46	<0.001	0.001	0.08
VAS scores (in maximum flexion position)	preoperative	24h after operation*	48h after operation *	72h after operation
First TKA	76.9±6.5	78.7±6.8	75.2±6.7	69.3±6.5
Second TKA	76.8±8.7	83.0±6.7	77.9±7.0	70.4±7.0
P values	0.938	<0.001	0.002	0.178

In addition, a subgroup analysis in this study was carried out to compare the effect of different intervals between the second and first knee on the difference in pain severity (ΔVAS = VAS scores in the second knee—VAS scores in the first knee). The patients were divided into 3 groups based on the different time intervals: less than 6 months, 6–12 months, and more than 12 months. General information of these 3 groups is listed in Table 3. Results of ANOVA and followed LSD-t test showed that the ΔVAS (both in rest position and in maximum flexion position) in the less than 6 months group was significantly higher than in the more than 6 months group, and there was no difference in ΔVAS between the 6–12 months group and the more than 12 months group (detailed statistical results are shown in Table 3).

**Table 3:** Comparison of ΔVAS in different time intervals.

Item	Less than 6 months (n = 26)	6–12 months (n = 34)	More than 12 months (n = 27)	F/*χ* ^2^	P
Age	68.2±9.4	68.3±8.5	68.6±7.6	1.187	0.288
Gender (male / female)	9/17	13/21	9/18	0.1724	0.9174
ΔVAS in rest position	13.0±12.5*,#	6.6±11.1	5.6±9.2	333.167	<0.0001
ΔVAS in maximum flexion position	6.3±6.3*,#	3.5±5.3	3.2±5.4	48.207	<0.0001

* Statistically significant between less than 6 months group and 6–12 months group (LSD-t test).

* Statistically significant between less than 6 months group and more than 12 months group (LSD-t test).

## Discussion

There is a controversy on the issue of choosing simultaneous bilateral TKA or staged bilateral TKA for patients in need of bilateral TKA. Investigators in favor of simultaneous bilateral TKA believe that it can reduce the cost of health care, shorten patient’s recovery time, and help patient get better knee functions without increasing the incidence of complications and mortality. However, investigators in favor of staged bilateral TKA insist that simultaneous bilateral TKA are associated with a longer operation time, anesthesia time, and extended tourniquet usage, which will increase the incidence of postoperative thrombosis, cardiovascular disease and mortality, etc.; whereas staged bilateral TKA may reduce these risks and achieve a more favorable prognosis [24–27].

Currently, most studies have been focused on the clinical evaluation of the above-mentioned simultaneous bilateral TKA and staged bilateral TKA, but very few studies have compared the difference between the results of the two surgery in staged bilateral TKA. Therefore, this study focused on the comparison of the early postoperative pain in the first and second knee in staged bilateral TKA. Research showed that early severe postoperative pain occurred mainly within 3 days after operation, and sleep quality in about 44–57% of patients was affected due to the postoperative pain within 3 days after the surgery [20]. Meanwhile, sleep deprivation can in turn reduce pain tolerance threshold and increase the severity of pain, therefore, pain relief within 3 days after operation is very important. The pain management protocol for initial knee replacement is often less effective when used for pain relief in the second knee, so that surgeons usually increase the dosage of analgesic after the second operation. However, this practice is due to lack of clinical evidence. This study focused on the comparison of the VAS scores in the first and second knee in staged bilateral TKA at 24h, 48h and 72h after operation. The results showed that, for patients receiving staged bilateral TKA, the VAS scores in the second knee were significantly higher than the first knee within 48h after operation, including ΔVAS in rest position and ΔVAS in maximum flexion position; but there was no significant difference at 72h as the ΔVAS decreased gradually. The two operations utilized the same surgical technique, the same implants, the same analgesic solutions and the same rehabilitation strategies, so the pain stimuli induced by the two operations were considered to be similar. Kim [28] suggested that the significant difference in early postoperative pain in staged bilateral TKA may be attributed to a hyperalgesia state in those patients, which is defined by the International Association for the Study of Pain (IASP) as a state that a general pain stimulus may produce a more intense pain [20]. He believed that this hyperalgesia state may be induced by the first knee operation, and result in pain allergy for the contralateral knee. Woolf [29, 30] believed that the generation mechanism of such hyperalgesia is evoked by the neural remodeling and central sensitization which is induced by harmful pain stimuli coming from tissues (such as skin, muscles, nerves, and connective tissues) around the operated joints and may affect subsequent neuromodulation processes. Suzuki [31] and Wieseler-Frank [32] also mentioned in their studies that the persistent pain after the initial surgery can change the body’s way of managing the pain and sensitize the cortical structures and ultimately result in central sensitization of the pain. However, some studies have shown that patients with severe osteoarthritis have already reached a hyperalgesia state due to long-term pain stimuli [33, 34]. Therefore, more research is needed to confirm whether the difference in postoperative pain severities in the two operations was caused by operation-induced hyperalgesia.

Psychological factor is also one of the important factors that contributes to the difference in the severity of postoperative pain. Because of the nature of this study (a retrospective study), all enrolled patients who completed staged bilateral TKA, most of them chose our hospital to do the second TKA because of the good outcome of the initial TKA surgery, and these patients usually had greater expectations for the second operation than the first operation, so their postoperative pain may be amplified because of their higher expectations.

Moreover, some investigators believed that tolerance to opioids may be associated with the different severities of postoperative pain as well [35] because the long-term use of opioid analgesics will gradually decrease their analgesic effect. However, this conclusion is under debate. Goldstein [36] found in his animal studies that although tolerance exists in opioid analgesics, it can completely disappear after drug withdrawal for 48 hours. Yaksh [37] found that long-term use of morphine can gradually reduce its analgesic effect, which will return to normal after drug withdrawal for 7 days. Therefore, the feature of fast recovery of the tolerance to opioids indicates that tolerance is not the main factor for the difference in the severity of postoperative pain. However, some reports indicated that more than 36% of patients still use large doses of opioids for pain control 30 days after TKA [38]. With the latest AAOS guideline suggesting that opioid analgesics, such as morphine or tramadol, have a higher grade than NSAID analgesics for pain control and can be regarded as the drug of choice after joint replacement surgery, more and more patients will prefer opioids for postoperative pain control. If the time interval between the two operations is short, we suggest that morphine withdrawal one week before the second operation may reduce the postoperative pain.

The subgroup analysis in this study showed that different time intervals between the two operations in staged bilateral TKA have certain impact on the changes in early postoperative pain (24h after operation). The ΔVAS in the less than 6 months group was significantly higher than in the more than 6 months group, and there was no significant difference in ΔVAS between the 6–12 months group and the more than 12 months group. Nevertheless, intra-group comparison of the three groups showed that: VAS scores after the second operation in each group were still significantly higher than those after the first operation. Therefore, we believed that as the extension of the time intervals between the two operations increased, the hyperalgesia state induced by the first operation can be gradually reduced. The early postoperative pain level after the second operation in patients with an interval of more than 6 months was significantly lower than those with an interval of less than 6 months, while there was no significant difference between patients with an interval of 6–12 months and an interval of more than 12 months.

There were also some limitations in this study. Firstly, this was a retrospective controlled study with biases and insufficiency in its evidence level compared with randomized controlled clinical studies. However, by using strict inclusion and exclusion criteria, we can effectively minimize the biases in this study. Secondly, more controlled subjects should be included in the study in order to improve the level of evidence. In TKA patients, women usually have greater postoperative pain than men [39], which means a high proportion of women may have influenced the results. In this research, the proportion of female patients was higher but not significant. The ratios of female and male in the 3 subgroups were also not significantly different. So gender may not have played an influential role. In addition, there were other limitations, such as the small sample size and the large time interval range between subgroups, which may compromise the strength of recommendations. To address the limitations in this study, we are planning to carry out an RCT research with a larger sample size and a more rational design to confirm and consolidate our conclusions. However, the significant difference in VAS scores between the first and second knee within 48 hours after operation mentioned herein can still suggest that the second TKA is indeed different from the first TKA; and the statistically significant differences in the subgroup analysis can also show that the change in postoperative pain in patients with an operation interval of more than 6 months is significantly lower than those with an interval of less than 6 months.

In conclusion, the severity of pain within 48 hours after the second operation in staged bilateral TKA was significantly higher than that after the first operation, and different time intervals between the two operations may also have had an impact on the changes in the early postoperative pain since the ΔVAS in the group of less than 6 months significantly higher than that in the group of more than 6 months. These results may provide effective clinical evidence to enhance the analgesic strategy in the second operation in staged bilateral TKA. And for the management of postoperative pain in staged bilateral TKA, it’s better to recommend that the interval between two operations should be more than 6 months, which may alleviate postoperative pain in the second knee, improve patient satisfaction, and speed up patient‘s rehabilitation process.
